# Wound Stress, an Unheeded Factor for Echinacoside Accumulation in *Cistanche deserticola* Y. C. Ma

**DOI:** 10.3390/molecules23040893

**Published:** 2018-04-11

**Authors:** Gaosheng Hu, Tianran Wu, Yue Chang, Xinyi Zhan, Jingming Jia

**Affiliations:** School of Traditional Chinese Materia Medica, Shenyang Pharmaceutical University, Shenyang 110016, China; wtr_00@163.com (T.W.); cy1796478564@163.com (Y.C.); z15202014102@163.com (X.Z.)

**Keywords:** biosynthesis, *Cistanchedeserticola* Y. C. Ma, echinacoside, phenylalanine ammonia lyase, physical wounding

## Abstract

*Cistanche deserticola* Y. C. Ma, a precious parasitic medicinal herb distributed in desert areas in the Northwest of China, also known as “desert ginseng”, has been used in China for thousands of years for its nourishing effects. The phenylethanoid glycosides (PeGs) have been proven as the main effective compounds due to their neuroprotective effects and were used for quality control. In this study, echinacoside content, a representative PeG, total phenolic content, DPPH scavenging activity, and PAL activity were determined in different tissues of *C. deserticola*. Our results showed that most indices had a similar pattern of scale > cambium ring > pith and bottom part > middle part > upper part. Besides, stereomicroscopic observation showed that the scale surface was densely covered with physical wounds formed during vertical and broadwise growth in sand. Thus, wound area was quantified and a linear regression analysis was conducted between wound area and PAL activity, total phenolics, and echinacoside content. Our results suggested that physical wounding caused by sand might play an important role in echinacoside biosynthesis which has never been noticed in *C. deserticola* development. Furthermore, the coexistence of the highest PAL activity and highest echinacoside accumulation in scale tissue might indicate that the biosynthetic site of echinacoside in *C. deseticola* Y. C. Ma is mainly in the scale tissue.

## 1. Introduction

*Cistanche deserticola* Y. C. Ma, a traditional Chinese medicinal herb, has been used for centuries in China for its nourishing effects. It is a perennial succulent plant and a parasitic medicinal herb belonging to the *Cistanche* genus of the Orobanchaceae family. The plant grows in desert areas of Northwest China and is a holoparasite on the roots of *Haloxylonammodendron*. It is usually harvested for medicinal use in spring before it grows out of the sand in the autumn season. The purification of its chemical constituents and structural elucidation studies of *C. deserticola* began in the 1980s and until now, 17 phenylethanoid glycosides (PeGs) have been isolated from *C. deserticola* [1] among which, echinacoside is the most abundant PeG and is used for the quality control index in China Pharmacopoeia [2]. The demonstration of PeGs as effective compounds in protecting neuronal cells from damage [3,4,5,6] caused by chemicals and aging has driven the increase in market demand for commercially available health foods and drugs.

Due to overharvesting, corruption of habitat, and complex parasitism, the natural resources of *C. deserticola* are on the edge of extinction and, in 2003, it was listed in CITES (The Convention on International Trade in Endangered Species of Wild Fauna and Flora). In order to maintain sustainable utilization of this precious material, artificial cultivation and cell suspension culture were carried out mainly in China by several research groups [7,8,9,10]. Until now, the artificial cultivation has been successful in Ningxia and Inner Mongolia provinces, where techniques have been developed to provide cultivated materials of equal quality to wild ones [11]. The cell suspension culture provides a useful model for research of PeGs accumulation and biosynthetic pathways. However, few reports have been published on the quality formation mechanism of wild or cultivated *C. deserticola*, and, more specifically, on the effects of growing environment on the PeGs biosynthetic enzymes activity or transcriptional level. Besides, the biosynthetic site of echinacoside has also not been fully described.

As we know, PeGs are phenylpropanoid compounds, which are reported as phytoalexins in many plant species against biotic and abiotic stresses such as physical wounding, microbe infection, drought, sanity, UV, and heavy metal ion exposure [12]. As this fleshy plant grows integrally in sand underground during most of its lifetime, we investigated whether physical wounding from sand friction during broadwise and vertical growth underground can be a major factor in the quality formation of *C. deserticola*. Phenylalanine ammonia lyase (PAL), catalyzing the conversion from l-phenylalanine to *trans*-cinnamic acid, has been proved as the key enzyme in phenylpropanoid biosynthesis and a connection step between primary and secondary metabolism in plant species [13]. PAL up-regulation resulted in significant accumulation of lignin, flavonoids, and proanthocyanidins which provide tissue strengthening, antioxidant, and anti-feedant protection in stressed tissues [14].

In this study, we determined the contents of echinacoside, total phenolic compounds, DPPH scavenging activity, and PAL activity in different tissues from different parts. Besides, the scale surface characteristics of *C. deserticola* were reported for the first time and the possible relationship between surface characteristics and PAL activity, total phenolic compounds, and echinacoside content were discussed.

## 2. Results and Discussion

### 2.1. Content Determination of Echinacoside and Total Phenolic Compounds

**Figure 1:**
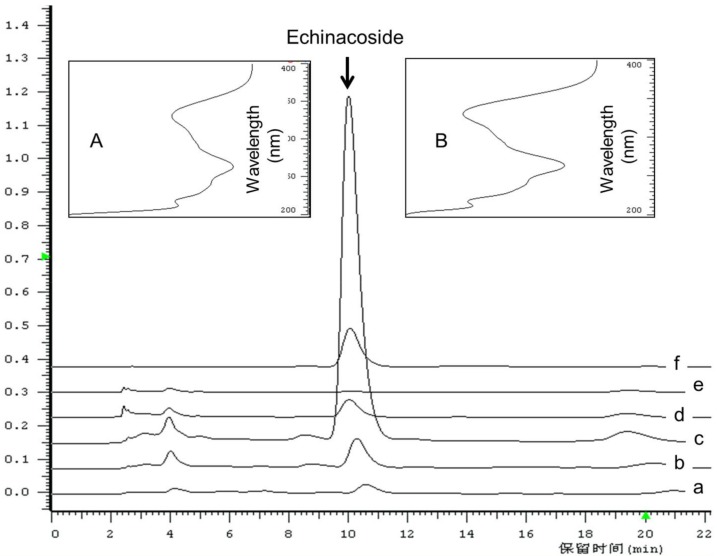
UV spectrum of echinacoside standard (**A**) and corresponding peak with same retention time in bottom scale tissue (**B**); RP-HPLC chromatogram of methanol extract from top scale (a), middle scale (b), bottom scales (c), bottom cambium ring (d), bottom pith (e) of *C. deserticola* and Echinacoside standard (f).

**Figure 2:**
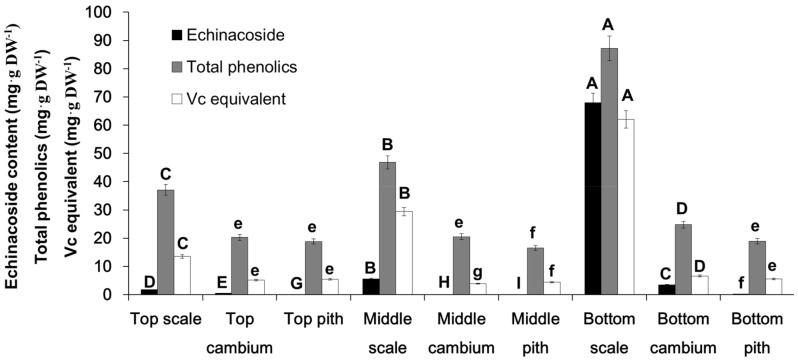
Content determination of echinacoside, total phenolics, and Vc equivalent in different tissues and parts of *C. deserticola*. Letters above the columns represent significance (A–I: *p* < 0.01; a–h: *p* < 0.05).

Echinacoside was identified after comparison of the retention time and UV spectrum recorded in the HPLC-DAD analysis, as shown in Figure 1A,B, according to which, the UV spectrum showed specific and identical absorption wavelength. Under current HPLC conditions, echinacoside was well separated from other interfacing peaks, which suggested that the analytical condition is suitable, and echinacoside is the main PeGs compounds in *C. deserticola*. In Figure 2, the contents of echinacoside, total phenolic compounds, and DPPH scavenging activity (expressed as Vc equivalent) of different tissues from three parts were shown that, in same part, the order of echinacoside, total phenolics, Vc equivalent were all scale > cambium > pith (except in top tissues, in which, total phenolic and Vc equivalent were not significantly different in cambium and pith tissue). When compared with the same tissue from different parts of the plant, in the case of scale tissue, which accumulated the highest content of the determined compounds, the content order of echinacoside, total phenolics, and Vc equivalent is bottom part > middle part > top part. However, in the case of cambium and pith tissue, there is not such a clear pattern, except that three indexes were highest in the bottom part.

### 2.2. DPPH Scavenging Capacity of Methanol Extract from Different Tissues

According to the reported literatures, the neuroprotective mechanism of echinacoside and other PeGs is mainly due to their antioxidant activity [3,4,5,6]. Therefore, the DPPH scavenging activity of methanol extract from different tissues was compared using Vc equivalent (mgVc·g DW^−1^). As indicated in Figure 2, the Vc equivalent showed a similar pattern to total phenolic content. This result also demonstrated that scales are an essential source for the antioxidant activity of *C. deserticola*. Our result provided the validity for the requirement in the China Pharmacopoeia (2015 Edition) [2] that the medicinal part of *C. deserticola* should be fleshy stem with scales.

### 2.3. PAL Activity in Different Tissues from Different Parts

**Figure 3:**
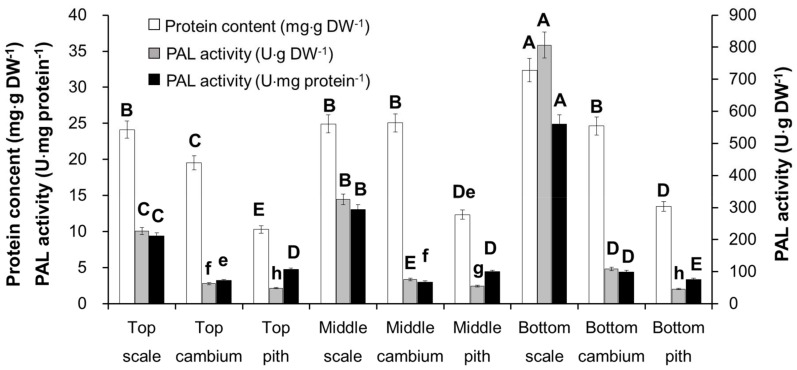
PAL activity and total protein content determination in different tissues and different parts of *C. deserticola*. Letters above the columns represent significance (A–I: *p* < 0.01; a–h: *p* < 0.05).

There are two main groups in PeGs, coumaroyl, caffeoyl or feruloyl, and phenylethanol or its deritives. The former is derived from l-phenylalanine [15] and the latter is reported to be derived from tyrosine through l-Dopa or dopamine, which has been proven using an isotope labeled experiment in *Olea europaea* cell suspension culture [15]. Besides, it was hypothesized that the phenylethanol group can also be derived from phenylalanine through phenylpyruvate or phenylethylamine, which is concluded from transcriptome analysis of *C. deserticola* [16]. In this study, PAL activity was assayed in different tissues from different plant parts. The unit of content determination of echinacoside and total phenolic content was mg·g DW^−1^. However, in the PAL activity assay, the same amount of crude protein was used in the reaction. According to the protein concentration determination results, we found that the protein concentration in different tissues differed significantly, as shown in Figure 3. Protein contents in scale and cambium tissue were much higher than that in pith tissue, which will cause bias in regression analysis. Therefore, combined with the protein content and PAL activity assay result, total PAL activity in same amount of dried biomass of different tissues was calculated as U·g DW^−1^. Most importantly, these results indicated that in scale tissue, PAL activity showed a similar pattern to total phenolic content, echinacoside, and Vc equivalent: bottom scale > middle scale > top scale, and in same part, scale tissue > cambium tissue > pith tissue.

**Figure 4:**
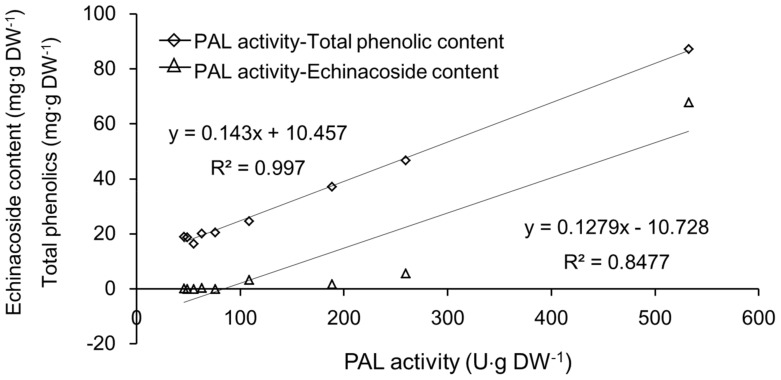
Linear regression analysis between PAL activity and echinacoside, total phenolic content.

Based on these results, linear regression analyses between total PAL activity and echinacoside and total phenolic content were carried out. As indicated in Figure 4, the correlation coefficient (R^2^) between total PAL activity and echinacoside content was 0.848, and R^2^ between total PAL activity and total phenolic compounds content was 0.997. In our previous report [17], the transcriptional level of the CdPAL-1 gene showed a high correlation coefficient with total phenolic content in different parts of *C. deserticola*. As we know, PAL belongs to a gene family, and there are usually several PAL genes in plant species. This has also been proved in RNA-seq analysis of *C. deserticola* [16]. Thus, in this study, the total PAL activity was assayed in nine parts from three plants mentioned in section 3.2. The co-existence of the highest total phenolic content, echinacoside, and PAL activity in scales can be considered as a proof that the biosynthetic site of echinacoside is mainly the scale tissue of *C. deserticola*. It was reported that, in *C. tubulosa*, the echinacoside content in the haustorium phloem was higher than in the xylem and whole *C. tubulosa* plant and it was concluded that the biosynthetic site might be haustorium phloem tissue [18]. However, the scale tissue was not separated and determined, and the scale surface characteristics were not mentioned. Another similar result has been reported in *Salviae miltiorrhizae* where the tissue accumulation of tanshinone diterpenoids was mainly in the root periderm, and transcriptome analysis showed significantly high expression levels of genes involved in the terpenoid biosynthesis pathway compared with phloem and xylem tissue [19]. However, the reason why the root periderm accumulated the highest level of tanshinone diterpenoids was not discussed. Our result provides a possible mechanism for the accumulation of secondary metabolites in tissues that contact soil or sand and, consequently, the microbes inside.

### 2.4. Microscopic Observation

**Figure 5:**
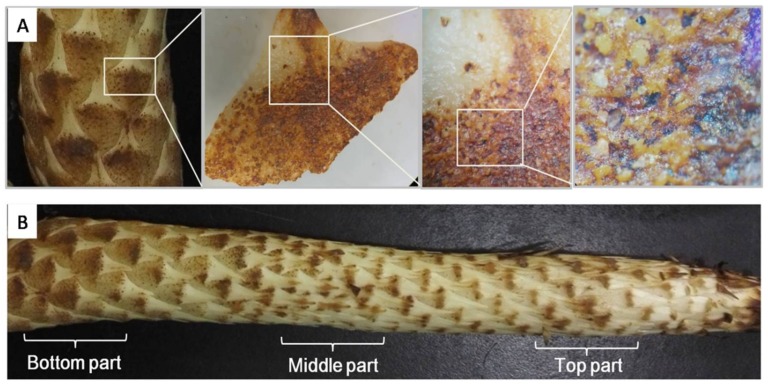
Stereomicroscopic vision of scale surface (**A**) and partition illustration (**B**) of *C. deserticola.*

**Figure 6:**
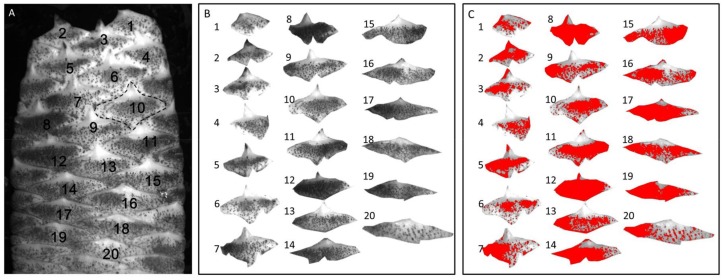
Scale numbering (**A**), scale matting (**B**), and wounding area quantification (**C**) of *C. deserticola.*

To find the reason why PAL activity was so much higher in scales than in other tissues, the scale surface characteristics were observed using a stereomicroscope. The result showed that scale surface was densely covered with physical wounds caused by sand friction during underground growth (Figure 5A and Figure 6). As reported, PAL activity in other plants species can be up-regulated by physical wounding, and results in higher accumulation of phenolic compounds like lignin, proanthocyanidin, and flavonoids and in functions, such as cell wall strengthening, anti-feedant, and antioxidant agents, respectively [20,21]. Interestingly, it is easy to see with the naked eye that there are much more serious wounds on scales from the bottom part than middle and top parts (Figure 5B). Therefore, we investigated whether the wounding area could be quantified and related with PAL activity.

**Figure 7:**
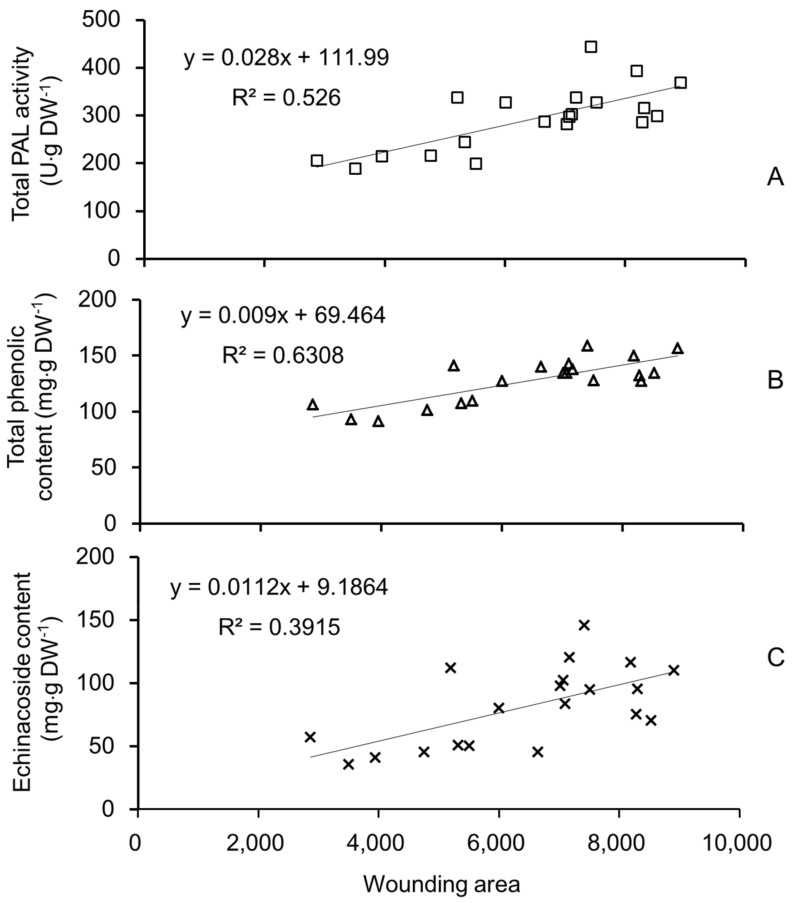
Linear regression analysis between wounding areas and PAL activity (**A**), total phenolic content (**B**), and echinacoside (**C**).

Linear regression analysis between quantified wounding area and PAL activity, total phenolic compounds, and echinacoside was carried out. As shown in Figure 7, the R^2^ between wounding area and PAL activity was 0.526 (*r* = 0.7253, *df* = 18, *p* < 0.01), the R^2^ between wounding area and total phenolic compounds was 0.6308 (*r* = 0.7942, *df* = 18, *p* < 0.01), and the R^2^ between wounding area and echinacoside was 0.3915 (*r* = 0.6257, *df* = 18, *p* < 0.01). From the table of critical value for significance test [22], when *p* < 0.01 and *df* = 18, the critical *r* value is 0.5614. These results demonstrated that physical wounding area is highly correlated with PAL activity, total phenolic content, and echinacoside content in scales of *C. deserticola.* Considering that 17 PeGs have been isolated from *C. deserticola*, the PeGs other than echinacoside might also account for the considerable content in *C. deserticoa* scales, and with more compounds determined, the coefficient might be higher. However, due to the lack of other standard PeGs, the contents of other PeGs were not determined.

In this study, it was observed that the PAL activity, echinacoside content, and total phenolic content showed similar patterns, and that the correlation coefficient (R^2^) between PAL activity with echinacoside and total phenolic content were 0.847 and 0.997, respectively, which meant that PAL played an important role in echinacoside and total phenolic content biosynthesis. Besides, the coexistence of the highest PAL activity and echinacoside content might indicate that the biosynthetic site of echinacoside is mainly in the scale tissue.

As a holo-parasitic plant, the water and nutrients required for its growth are acquired from the host plant. Its leaves transition from white and triangular to brown scales covering whole plant surface. Thus, the scales are the main tissue contacting the surrounding hard sand particles. During underground growth, the plant will face serious friction caused by its expansion and elongation, which causes numerous physical wounds, as observed by stereomicroscopy of scales. According to the growth characteristics of *C. deserticola*, the bottom part usually has the highest diameter (Figure 5) and is formed at the earliest point after germination, meaning that the scales of the bottom part have faced the highest pressure for the longest duration. Furthermore, the wound areas of individual scales were quantified and showed a co-relationship with PAL activity, total phenolic content, and echinacoside content, and the correlation coefficients were 0.526, 0.630, and 0.391, respectively. These results partially proved our hypothesis that during underground growth, sand particles surrounding *C. deserticola* cause physical wounding on the scales covering the plant surface, which brings higher risk of microbial infection and triggers the plant’s defense mechanisms, ultimately resulting in higher accumulation of total phenolic content and echinacoside, and playing an important role in the quality formation of *C. deserticola*. Our results will facilitate the discovery of genes involved in PeGs biosynthesis in *C. deserticola* and these findings might also translate to other desert growing Orobanchaceae plants, such as *Cynomorium songaricum*, *C. salsa,* and *C. tubulosa,* and even provide important clues for the quality formation process of medicinal herbs with the root as the medicinal part. However, illustration of the detailed mechanism of physical wounding on echinacoside biosynthesis requires further research. The role of microbial infection caused by sand wounding should also be considered.

## 3. Materials and Methods

### 3.1. Chemicals and Reagents

Echinacoside was purchased from China Institute of Food (city) and Drug Control with purity over 95%. PAL activity assay kit was purchased from Nanjing Jiancheng Institute of Biological Engineering, Nanjing, China. HPLC grade methanol was purchased from J & K Chemicals. Vitamin C and DPPH was purchased from Sigma-Aldrich, Shanghai, China. Analytical grade methanol, 95% ethanol, and formic acid were purchased from Yuwang chemical Group, Weifang, Shandong province, China. All data presented are expressed as the average of three replicates with standard error bars as indicated.

### 3.2. Plant Sample Collection and Partition

Fresh plant samples were collected in Alashan Zuoqi areas of Alashan, Inner Mongolia province, China in 11 October 2016 and were authenticated as *C. deserticola* Y. C. Ma, by associate professor Jia Lingyun in School of Traditional Chinese Materia Medica, Shenyang Pharmaceutical University. The specimen was given a voucher number of SPU20161526-29 and was stored in Herbarium of Shenyang Pharmaceutical University.

The bottom, middle, and top parts of three plants with similar height and surface characteristics were sampled and divided into three layers; scales, cambium ring, and pith. Each tissue from different parts were lyophilized until constant weight was observed under −50 °C. Lyophilized materials were ground into fine powders, passed through 60 meshes, and stored in −80 °C for future analysis.

For the quantification of wounding area, the scale layer from another *C. deserticola* plant sample was cut off and pictured following quick freezing using liquid nitrogen and was lyophilized at −50 °C until constant weight was observed. Wounding area was quantified as described in section 3.9. Individual scales were cut off from lyophilized scale layer and grounded into fine powder. 30 mg of powder was used for methanol extract preparation and content determination of echinacoside and total phenolic content was conducted as described in materials and methods section. Meanwhile, 20 mg of powder was used for crude protein extraction and PAL activity assay as described in section 3.7.

### 3.3. Extract Preparation

30 mg powder of each tissue from three different parts were weighed and added to a 1.5 mL Eppendorf tube containing 1 mL methanol which was then vortexed and sonicated at room temperature for 45 min, followed by centrifugation at 12,000 rpm for 10 min. The supernatant was transferred to new tubes and filtered through 0.45 µm filters, ready for echiancoside, total phenolic content, and DPPH scavenging activity assays.

### 3.4. Content Determination of Echinacoside Using RP-HPLC

A Hitachi HPLC (Tokyo, Japan) system coupled with DAD detector (L-2420), auto-sampler (L-2200), column oven (AT-330), and reverse phase column (Waters, MA, USA, XSELECT™ HSS C_18_, 4.6 × 250 mm, 5 µm) was applied for echinacoside content determination. The detection wavelength was set to be 330 nm according to the literature [23]. The elution condition was methanol-0.1% formic acid (30:70, *v*/*v*).

Standard compounds were accurately weighed and dissolved in 5 mL measuring flasks to final concentrations of 10.0 mg·mL^−1^. Serial dilutions were made for standard curve preparation. 10 µL of each standard solution dilution was injected into HPLC. Regression analysis between echinacoside concentrations (X) and peak areas (Y) were conducted using a Microsoft 2007 Excel software. The standard curve and linear range of echinacoside was Y = 6 × 10^6^X − 4193.9, (R^2^ = 0.9998, linear range: 0.0032 to 2.0 mg·mL^−1^). Peak areas in samples with corresponding retention time was recorded and used to calculate the concentration in extract obtained from the Extract Preparation section.

### 3.5. Total Phenolic Compound Determination Using the Folin–Ciocalteu Method

Ten µL of extract from different tissues of different parts were added to an Eppendorf tube containing 200 µL freshly prepared 2% sodium carbonate solution. The mixture was mixed vigorously and followed by a brief spin down. The mixture was incubated at RT for 5 min, and subsequently mixed with 10 µL 2× diluted Folin–Ciocalteu reagent. The final mixture was mixed vigorously and incubated at RT for 30 min. Absorption at 750 nm (A_750_) was measured in a microplate reader and recorded for calculation of total phenolic content [16]. The caffeic acid (Sigma-Aldrich, Shanghai, China) serial solution was used as standard compound and a standard curve between the amount of caffeic acid and A_750_ was prepared (Y = 0.8999X − 0.0078, R^2^ = 0.9973, linear range: 0.1 to 1.0 mg·mL^−^^1^) to calculate the equivalent total phenolic contents in different tissues from different parts.

### 3.6. DPPH Scavenging Activity Assay

Vitamin C (Vc) was dissolved in methanol and 100 µL series dilutions (0.01 to 0.08 mg·mL^−1^) were added to a 1.5 mL Eppendorf tube containing 600 µL DPPH (0.1 mg·mL^−1^), with methanol as control. After incubation at dark at room temperature for 30 min, the absorption at 517 nm (A_517_) were monitored in a microplate reader [24]. DPPH scavenging rate was calculated as follows: Scavenging rate (%) = (A_0_ − A_sample_)/A_0_ × 100%. A standard curve between Vc concentration (X) and DPPH scavenging rate (Y) was prepared: Y = 7.5907X − 0.0608, R^2^ = 0.9967, linear range: 0.010 to 0.080 mg·mL^−1^. Vc equivalent of sample extract was calculated using this standard curve and expressed as mgVc·g DW^−1^.

### 3.7. PAL Activity Assay

Crude enzyme extraction and PAL activity assay of samples was carried out using an activity assay kit following the manufacturers’ instructions. Protein concentration was determined using a Coomassie brilliant blue G250 method and BSA was used as standard. In each group, same amount of total protein was loaded in the reaction mixture containing l-phenyalanine as substrate. Standard curve of BSA was Y = 0.006X + 0.0175, R^2^ = 0.9917, linear range: 5.0 to 100.0 µg·mL^−1^, in which, Y represents A595 nm value and X represents BSA concentration (µg·mL^−1^). The total PAL activity was finally calculated based on the protein content determination and activity assay results as U·g DW^−1^ tissue.

### 3.8. Stereomicroscopic Visualization

A stereomicroscope (SMZ745T, Nikon, Tokyo, Japan) was used to visualize the surface characteristics. Fresh scales were placed on the sample platform with the surface facing upwards. White light was used during visualization.

### 3.9. Wounding Area Quantification

The scale layer of another fresh plant sample was cut off, pictured, and an image of each scale was matted and analyzed respectively, as shown in Figure 6, using the colony counting function of the Tianon image analysis software Tianon Colon Counter (v3.2, Tianon, Shanghai, China). The sensitivity was set to 37, under which most wounded regions can be recognized and the sum area of wounds were recorded.

### 3.10. Statistical Analysis

All experiments were repeated three times and data were represented as mean or mean ± SD. Significance analysis was carried out using the one-way ANOVA tool in Microsoft Excel. Letters above the columns in Figure 2 and Figure 3 represent significance (A–I: *p* < 0.01; a–h: *p* < 0.05).
